# Atomically precise metal nanoclusters meet metal-organic frameworks

**DOI:** 10.1016/j.isci.2021.103206

**Published:** 2021-09-30

**Authors:** Lianshun Luo, Rongchao Jin

**Affiliations:** 1 Department of Chemistry, Carnegie Mellon University, Pittsburgh, PA 15213, USA

**Keywords:** Catalysis, Nanoparticles, Nanotechnology, Nanomaterials

## Abstract

Significant progress has been made in both fields of atomically precise metal nanoclusters (NCs) and metal-organic frameworks (MOFs) in recent years. A promising direction is to integrate these two classes of materials for creating unique composites with improved properties for catalysis and other applications. NCs incorporated with MOFs exhibit an optimized catalytic performance in many catalytic reactions, in which MOFs play a vital supporting role or as cocatalysts. In this Perspective, we first provide a brief summary of the methods that have been developed for the preparation of NCs/MOF composites and the characteristics of these strategies are analyzed. Following that, some recent works are highlighted to demonstrate the crucial role of MOF matrices in the enhancement of NCs catalytic properties. Finally, we outline some potentially important aspects for future work. This Perspective is in hopes of stimulating more interest in the research on the integration of NCs with MOFs toward functional materials.

## Graphical abstract

**Figure:**
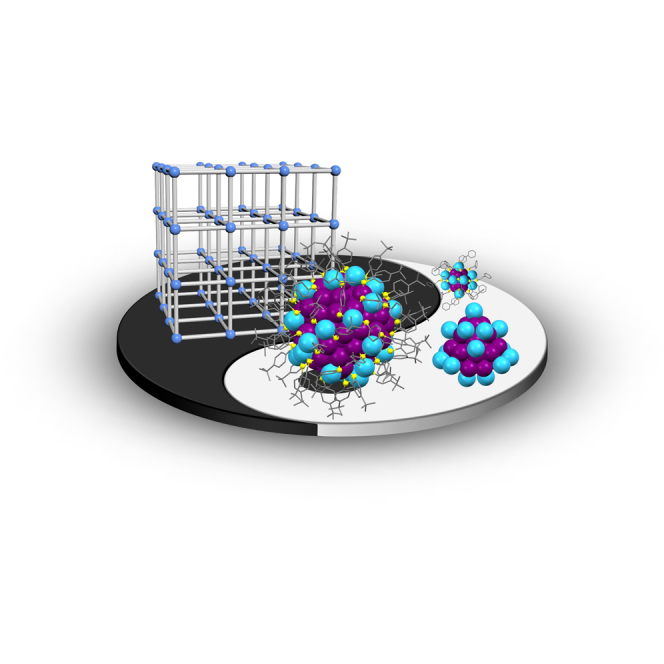

## Introduction

The past decade has witnessed the rapid development of atomically precise metal nanoclusters (NCs), including the synthetic strategies and prospective applications (Jin et al., 2016; Zhou et al., 2019). Particularly, NCs are of wide interest to the field of catalysis owing to their distinctive geometric and electronic structures (Du et al., 2020; Jin et al., 2021a). First, NCs possess the highest uniformity, precise atomic compositions, and well-defined atom-packing modes, allowing for structure-reactivity correlations (Li et al., 2021).In addition, exquisite control at the atomic precision (e.g., the atom type, quantity, and location, as well as the surface ligands) enables fine-tuning of the NC surface functionality to improve the catalytic properties (Jin et al., 2021a, 2021b).

However, two issues are posed in NCs-based catalysis: (1) the insufficient stability under harsh conditions (e.g., a tendency of aggregation) and (2) the steric hindrance of the ligands (e.g., for bulky reactants) (Li and Jin, 2013; Longo et al., 2020). The high surface free energy originated from the ultrasmall size not only gives rise to high catalytic activity but also often results in the aggregation of NCs, especially at high temperatures, which will cause a sharp decrease in active sites (Yan et al., 2018; Chen et al., 2019a). For the second problem, although surface ligands on NCs are necessary for protecting and maintaining the atomically precise structure, the existence of those ligands will reduce the accessibility of active sites by the increased steric hindrance (Li et al., 2018a; Shivhare et al., 2013; Fang et al., 2016). As a result, improving the catalytic performance requires figuring out how to stabilize NC structures while decreasing the shielding impact of ligands on metal sites.

To solve the problems, one of the potential strategies is to integrate NCs with metal-organic frameworks (MOFs). MOFs are a class of crystalline porous materials with well-defined pore architectures and tunable chemical functionalities (Zhou et al., 2012; Kirchon et al., 2018). Profiting from their unique features, MOF matrices can protect the incorporated guests from aggregation during the heterogeneous catalysis and also endow them with particular characteristics, such as the molecular sieving effects (Zhang et al., 2014). Furthermore, the electron transport and chemical bonding between the inserted guests and the porous frameworks are conducive to boosting the catalytic performance and expanding the reaction range (Huang et al., 2017a). So far, various nanosized guests have been incorporated into MOFs, such as metal oxide nanoparticles (Falcaro et al., 2016), metal nanoparticles (Yang et al., 2017; Pan et al., 2021), and quantum dots (Aguilera-Sigalat and Bradshaw, 2016). The integration of NCs with MOFs has progressed rapidly in recent years, and MOFs have demonstrated remarkable performance in overcoming the limitations of NCs in catalysis.

In this Perspective, we first summarize the reported methodologies for the fabrication of NCs/MOF composites, along with analyzing the advantages and limitations of those methods. Then, we introduce some recent work to illustrate how MOF matrices contribute to the NCs-catalyzed reactions. Finally, some perspectives are provided on the future developments in this field. We hope this Perspective will stimulate more research efforts toward this direction.

## Fabrication of NCs/MOF nanostructures

MOFs consist of two major components: the bridging organic ligands and the inorganic secondary building units (SBUs) comprising metal ions or clusters. The well-defined frameworks of MOFs are formed through the periodic connection between organic linkers and SBUs. In the last few decades, more than 20,000 MOFs have been reported (Fenlon, 2018). The hybridization of NCs with MOFs can be accomplished in several ways. On the one hand, NCs as guest species can be incorporated into the host framework of MOFs via three primary routes in terms of the synthetic approach: surface anchoring, pore-confined growth, and *in situ* encapsulation. NCs used as building blocks, on the other hand, can be coordinated with specific functional linkers to form the cluster-based MOFs, which we call the inter-nanocluster linking strategy. Here, it is worth mentioning that the few-atom clusters (e.g., Zr_6_(μ_3_-O)_4_(μ_3_-OH)_4_(COO)_12_) are not covered in this article (Rogge et al., 2017; Chen et al., 2019b). The NCs discussed below are typically composed of dozens of atoms in the core and are protected by thiolate (SR) and phosphine (PPh_3_) ligands.

**Figure 1:**
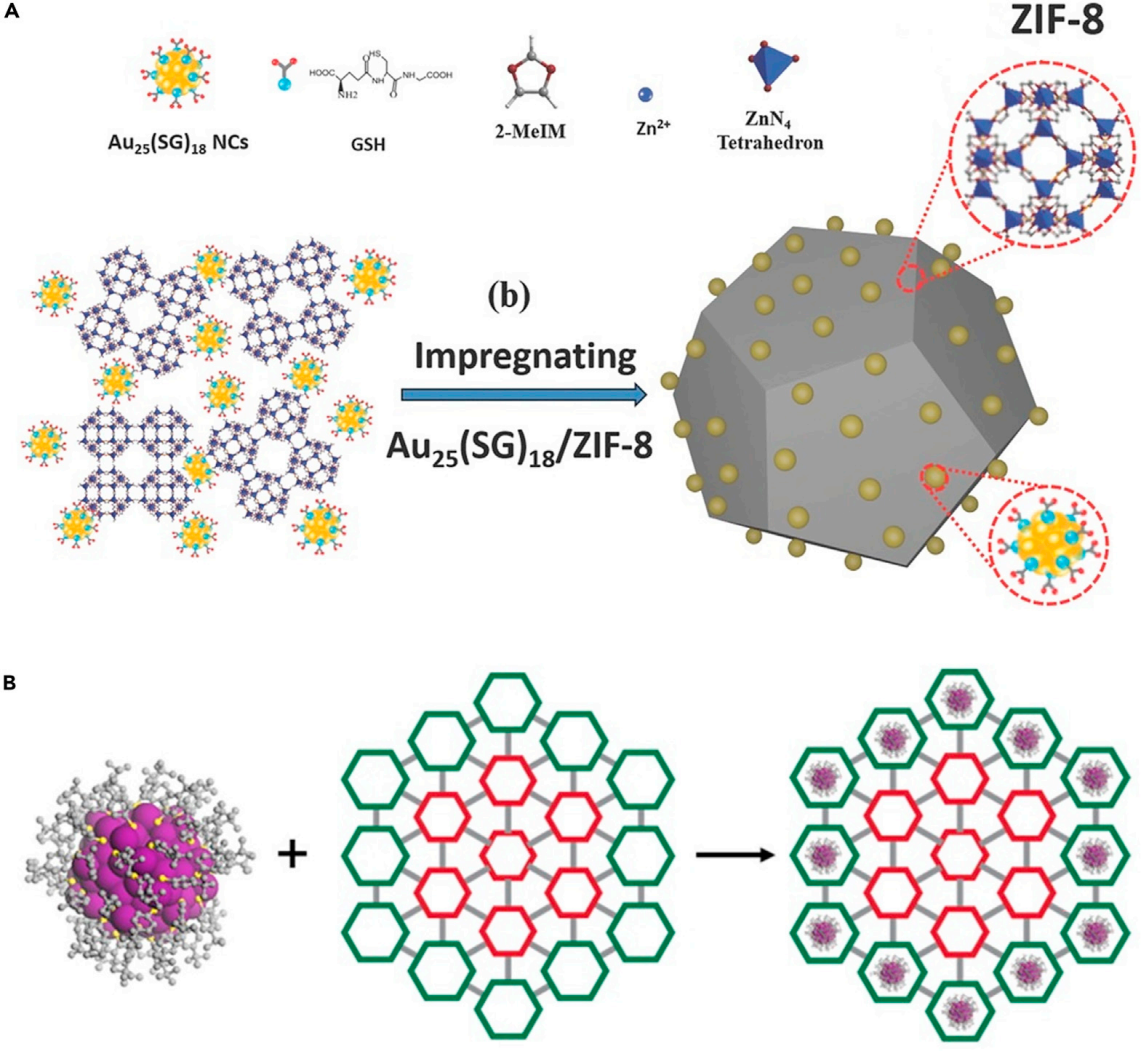
Construction of NCs/MOF composites (A) Schematic representation of the synthesis processes of Au_25_(SG)_18_/ZIF-8 (anchoring Au_25_(SG)_18_ on the outer surface of ZIF-8). Note: Au_25_ = 25-atom nanoclusters, SG = glutathione, ZIF-8 is a zeolitic imidazolate framework (ZIF) consisting of tetrahedrally coordinated Zn^2+^ ions connected by 2-methylimidazole linkers. Reproduced with permission from Luo et al. (2018). Copyright 2018 Wiley-VCH. (B) Schematic representation of Au_133_(SR)_52_ organization in the periphery of gradient bMOF-102/106. Reproduced with permission from Liu et al. (2016a). Copyright 2016 American Chemical Society.

Surface anchoring is the most straightforward method for combining NCs with MOFs, which may be accomplished using a simple wet impregnation process. Typically, mixing pre-synthesized MOFs and NCs in one pot can attach NCs onto the outer surface of MOFs, but there is a precondition that the size of NCs should be larger than the aperture size of MOF matrices (Figure 1A) (Luo et al., 2018). On the contrary, if the size of NCs is less than the MOF's aperture, NCs could easily diffuse into and be located in the interior channel of MOFs (Figure 1B) (Liu et al., 2016a). The driving force of surface anchoring strategy is the host-guest interactions between NCs and MOFs, such as the coordinative interaction and the electrostatic attraction. Anchoring NCs on the MOF surface can retard the migration and hence aggregation of NCs to some extent, as well as combining the properties of NCs and MOFs. Nevertheless, the NCs could be detached from MOF supports by washing due to the weak host-guest interactions, and it is also insufficient for the surface anchoring approach to eliminate the aggregation of NCs at harsh conditions (e.g., high temperatures). Thus, research on the integration of NCs with MOFs via surface anchoring is relatively less.

**Figure 2:**
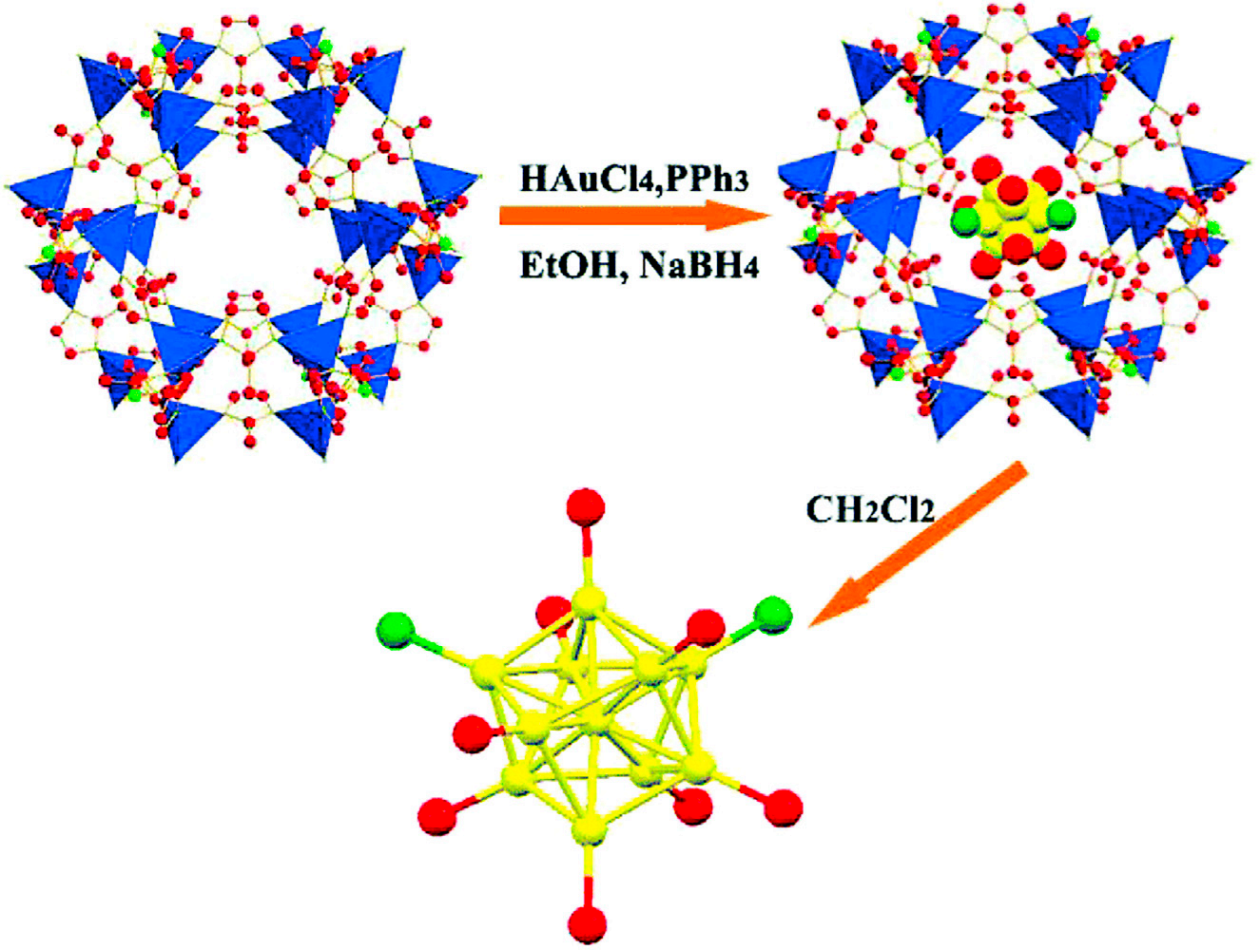
Schematic representation of the process of Au NCs synthesis in MOFs and the example of Au_11_@ZIF-8 Reproduced with permission from Liu et al. (2016b). Copyright 2016 Royal Society of Chemistry.

The growth of NCs within MOF pores is another viable strategy because the pore size of MOFs is highly tunable and can accommodate guests of various sorts and sizes (Deng et al., 2012). Pore-confined growth, as opposed to surface anchoring, is a more advanced immobilization technique that can give molecular-size selectivity to NCs while also protecting them against leaching and aggregation. In 2016, Liu et al. demonstrated the pore-confined growth by investigating the synthesis of Au_11_:PPh_3_ NCs inside ZIF-8 pores (Figure 2) (Liu et al., 2016b). After impregnating Au precursor into ZIF-8 and subsequent reduction by NaBH_4_, homogeneous Au_11_:PPh_3_ NCs were synthesized and uniformly disseminated in the framework of ZIF-8. Moreover, when MIL-101 with large pores was employed as the template, pure Au_13_Ag_12_:PPh_3_ NCs with a larger size than Au_11_:PPh_3_ can be obtained, manifesting the versatility of the pore-confined growth method. Since NCs are physically constrained in pores, the migration and aggregation of NCs can be effectively avoided at high temperatures. Thus, the ability of pore-confined growth to integrate and stabilize NCs inside MOF scaffolds is evident. Unfortunately, owing to the restricted pore size of MOFs, the sizes of NCs that can be produced may be limited. Furthermore, sometimes the pore-confined growth has difficulties in ensuring the atomic accuracy of NCs, as atomically exact NCs generally require kinetic control in the reduction stage, followed by a size-focusing step or separation procedure (Li and Jin, 2020; Liu et al., 2020; Chen et al., 2015).

**Figure 3:**
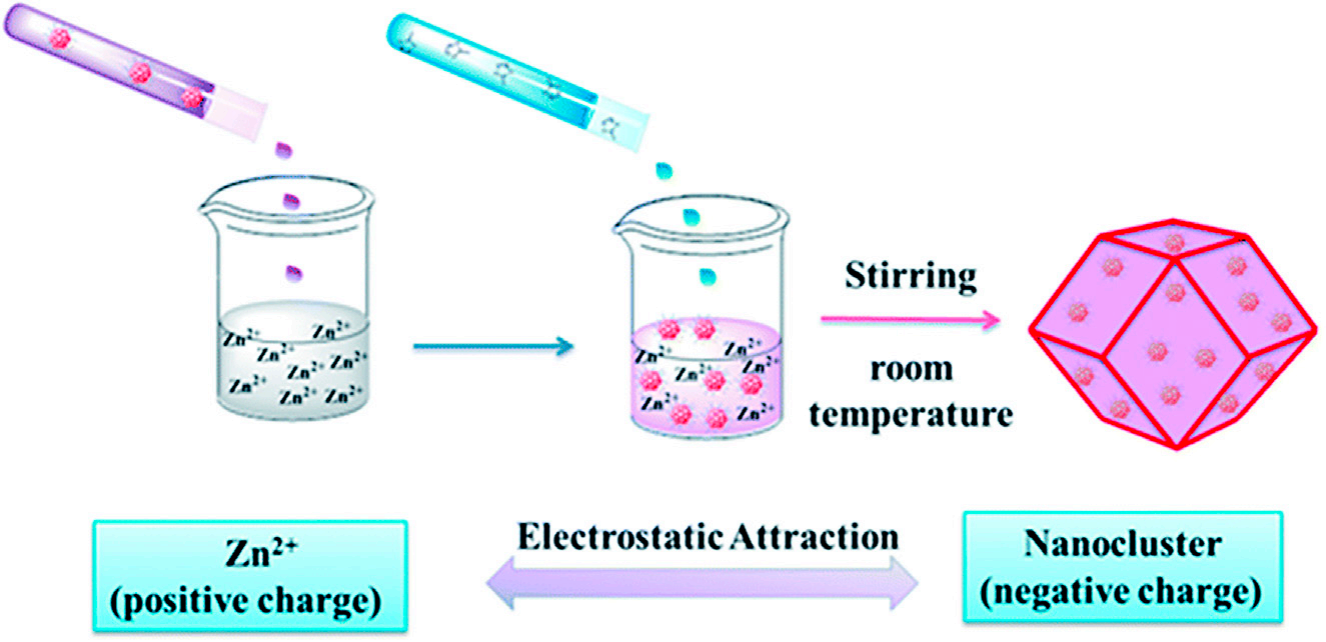
Schematic illustration showing the rational encapsulation of NCs@MOFs by electrostatic attraction Reproduced with permission from Sun et al. (2018). Copyright 2018 Royal Society of Chemistry.

*In situ* encapsulation, on the other hand, appears to be a more promising alternative with more possibilities. *In situ* encapsulation is to mix NCs and the precursors of MOF in one pot so that the NCs@MOF composites could cocrystallize during the synthetic process (Luo et al., 2018; Gao et al., 2018; Sun et al., 2018). Hence, this method allows any size and shape of NCs to be sterically encapsulated into MOFs. The tight MOF covering on the implanted NCs are like armor to protect NCs from migration and aggregation. In addition, the integrity of MOF porosity can be preserved since MOF is coated on the surface of NCs, facilitating the rapid diffusion of reactants within the MOF. Initially, coordination-assisted self-assembly was developed to encapsulate Au_25_(SG)_18_ into ZIF-8 (Luo et al., 2018), which takes advantage of the coordinating interaction between Zn^2+^ and the carboxyl group in -SG ligand of Au_25_ NCs, leading to the self-assembly of ZIF-8 surrounding Au_25_(SG)_18_ NCs. Later on, a more universal encapsulation strategy based on electrostatic attraction was established (Figure 3) (Sun et al., 2018). Taking Au_12_Ag_32_(SR)_30_@ZIF-8 as an example, the typical process includes (1) mixing anionic [Au_12_Ag_32_(SR)_30_]^4-^ NCs with a Zn^2+^ solution and (2) adding 2-methylimidazole into the [Au_12_Ag_32_(SR)_30_]^4-^/Zn^2+^ mixture. Then, [Au_12_Ag_32_(SR)_30_]^4-^ NCs can be uniformly encapsulated into ZIF-8 scaffolds without sacrificing the intrinsic porosity of ZIF-8. The electrostatic attraction between anionic NCs and positively charged metal ions is the driving force for successfully encapsulating NCs into MOFs. Accordingly, by rationally selecting NCs and MOFs, the electrostatic attraction-assisted encapsulation process is able to fabricate various NCs@MOFs composites, such as all the combinations of Au_12_Ag_13_, Ag_44_, and Ag_12_Cu_28_ NCs with ZIF-8, ZIF-67, and manganese hexacyanoferrate hydrate frameworks. All in all, *in situ* encapsulation is an elegant and versatile strategy for the integration of NCs with MOFs, which is capable of immobilizing NCs inside MOFs while preserving the intrinsic properties of both NCs and MOFs.

**Figure 4:**
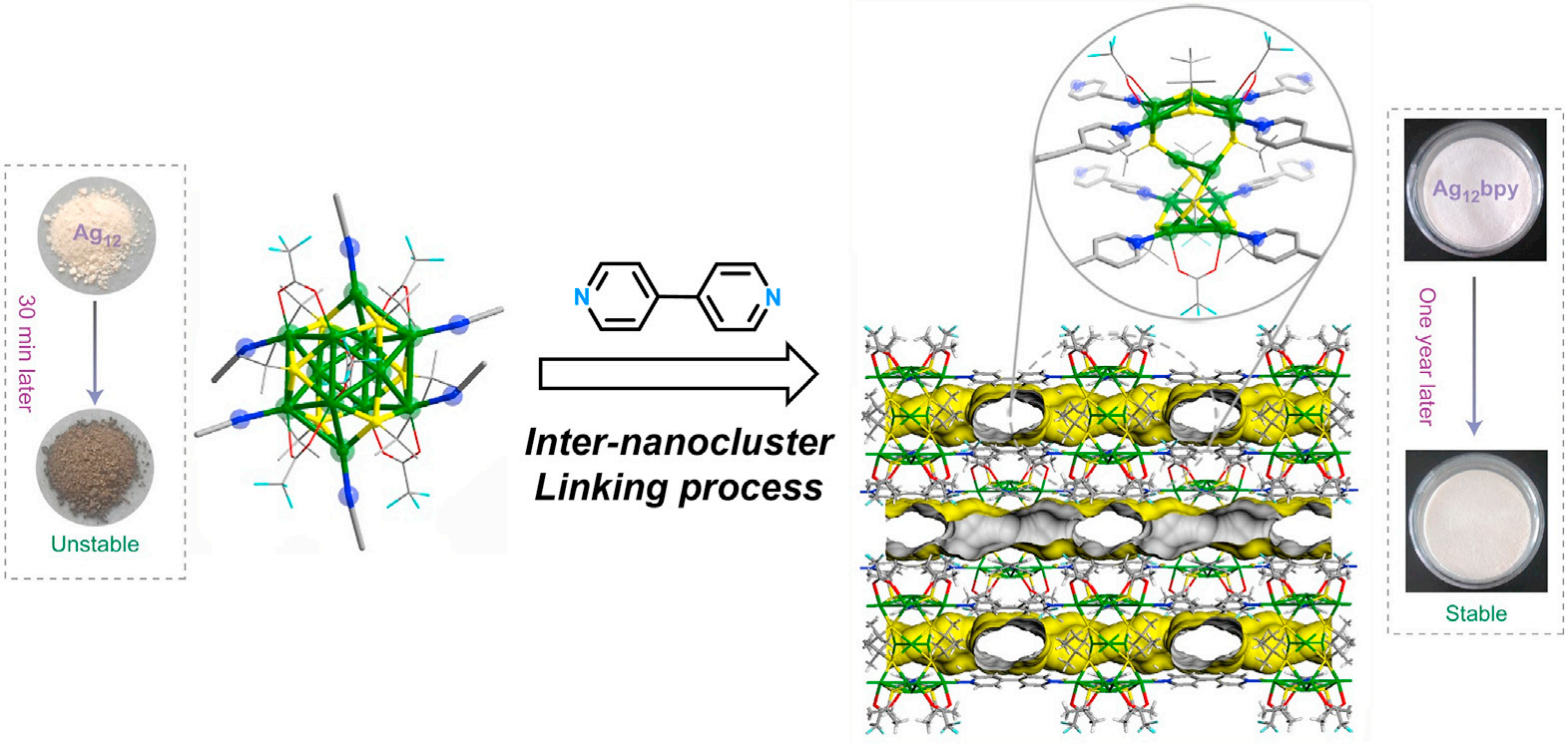
Schematic representation of self-assembly of Ag_12_ NCs building blocks into Ag_12_bpy porous frameworks Note: the yellow surface (on the right) represents the pore surface. Reproduced with permission from Huang et al. (2017b). Copyright 2017 Springer Nature.

Owing to the tailorable surface coordination chemistry of NCs, specific functional linkers can assemble NC building blocks into cluster-based networks. Such cluster-based networks possess unambiguous crystal structures and porous frame architectures, which are reminiscent of the characteristics of MOFs (Jin et al., 2021a; Kang and Zhu, 2019). The best-known examples of cluster-based MOFs are silver chalcogenolate cluster-based MOFs that rely on the Ag-N interactions. In 2017, Zang and co-workers demonstrated the inter-nanocluster linking of Ag_12_ NCs into the rigid Ag_12_-based MOFs (Ag_12_(S^*t*^Bu)_8_(CF_3_COO)_4_(bpy)_4_, designated as Ag_12_bpy below) by substituting the coordinated CH_3_CN ligands of Ag_12_ with the linear bidentate linker 4,4′-bipyridine (bpy) (Figure 4) (Huang et al., 2017b). In the framework of Ag_12_bpy, each [Ag_12_S_8_]^4-^ node is linked to four adjacent nodes via eight μ_2_-bpy bridges, and an open channel with a rectangle cross-section of dimensions 11.78 × 6.47 Å^2^ appeared. The inter-nanocluster linking process remarkably improved the stability of Ag_12_ NCs (from minutes to over 1 year), and a 60-fold enhancement in photoluminescence (PL, quantum yield from 0.2 to 12.1%) was observed after the assembly of Ag_12_ building blocks; thus, this strategy should be promising for extension to other systems by utilizing the functionalized NCs (Gunawardene et al., 2019; Li et al., 2016) for enhancing luminescence and catalytic functionalities (Takano et al., 2018; Higaki et al., 2018). The composites have been utilized in various applications (Huang et al., 2018; Du et al., 2018; Cao et al., 2019; Dong et al., 2020). Several other Ag NCs including Ag_8_ (Chang et al., 2018), Ag_10_ (Dong et al., 2018), Ag_14_ (Wang et al., 2018; Alhilaly et al., 2019; Deng et al., 2021), Ag_15_ (Alhilaly et al., 2019), Ag_18_ (Ma et al., 2019), and Ag_27_ (Zhao et al., 2020a) have also been constructed into two- or three-dimensional cluster-based MOFs using N-containing linkers with different configurations and functionalities. Indeed, the inter-nanocluster linking technique exhibited a distinctive superiority in the structural manipulation and property tailoring of NCs, allowing not only for the enhanced stabilization of NCs but also for the merging of functionalities of NCs and MOFs. However, one drawback is that the bridging linkers used to fabricate the cluster-based MOFs are limited to N-donor ligands only. Thus, novel bridging linkers or new types of bonding modes are still highly desired for enriching the family of the cluster-based MOFs.

## NCs/MOF nanostructures for catalysis

Unlike traditional nanoparticles, NCs possess several distinct features including their ultrasmall sizes, abundant unsaturated active sites, and large surface areas, all of which make them highly reactive in catalytic reactions. However, the higher tendency of aggregation and the lower exposure of metal sites of ligated NCs will reduce or even shut down the catalytic activity. Integrating NCs with MOFs is a feasible way to tackle these dilemmas, and the synergistic effect between NCs and MOF matrices can occasionally have unexpectedly favorable effects on catalytic performance. Here, we will discuss some recent work to demonstrate how the MOF matrices assist or participate in the NCs-catalyzed reactions.

**Figure 5:**
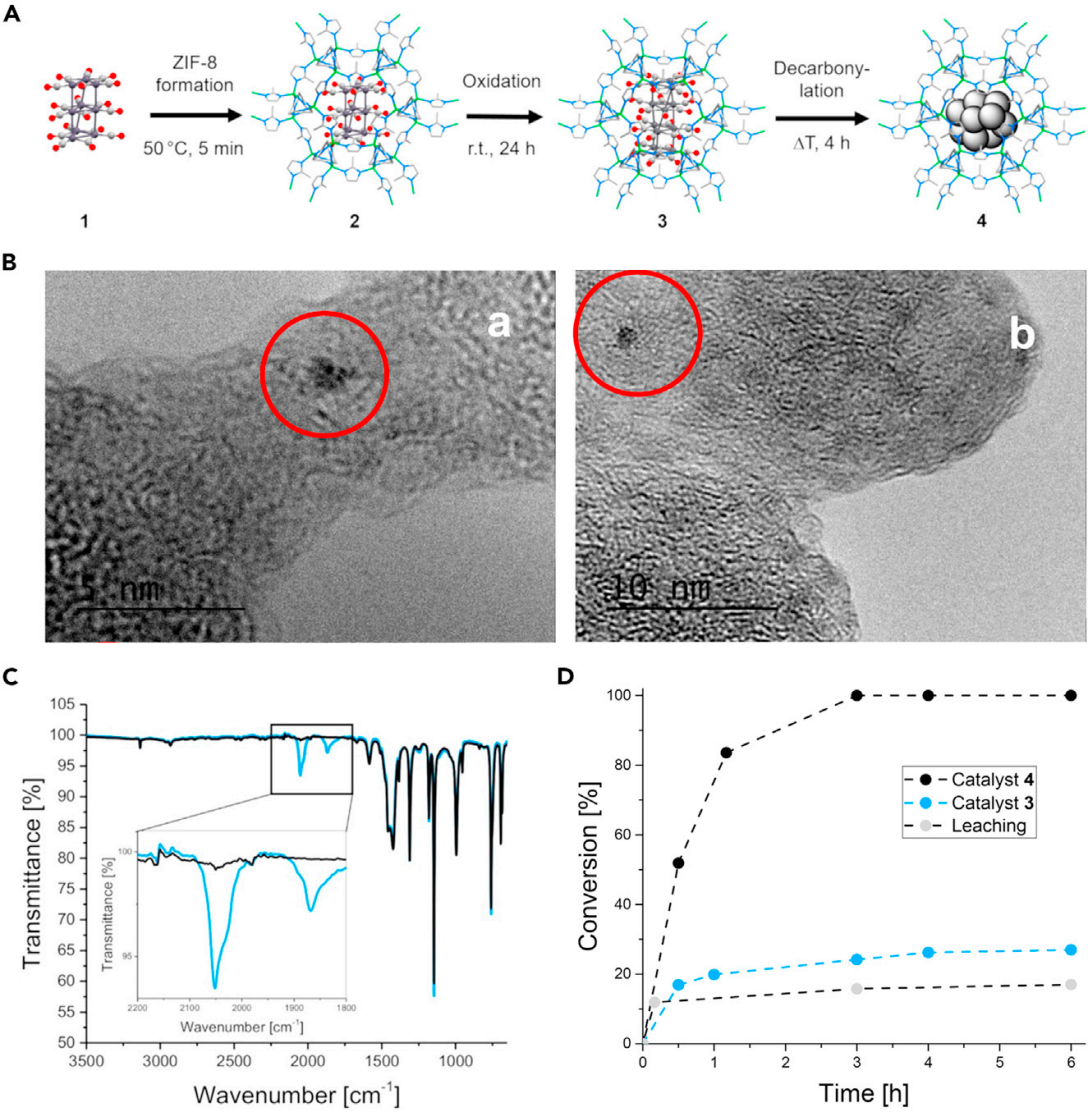
Characterization of PtNCs/ZIF-8 composites (A) Schematic representation of the synthesis of precise PtNCs in ZIF-8: (1) the Chini cluster [NBu_4_]_2_[{Pt_3_(CO)_6_}_3_], (2) [NBu_4_]_2_[{Pt_3_(CO)_6_}_3_]@ZIF-8, (3) [NBu_4_]_2_[{Pt_3_(CO)_6_}_4_]@ZIF-8, and (4) PtNC@ZIF-8. (B) High-resolution transmission electron microscopy (HR-TEM) images of 3 and 4: (left) a single PtNC in 3 (scale bar, 5 nm); (right) a single PtNC in 4 (scale bar, 10 nm). (C) Comparative FT-IR spectra of 3 (blue) and 4 (black). After heating under vacuum, the CO bands for terminal (2,051 cm^−1^) and bridging (1,868 cm^−1^) CO ligands (inset) disappeared completely, confirming the full decarbonylation. (D) Catalytic hydrogenation of 1-hexene with 3 and 4 as catalysts. Reproduced with permission from Kratzl et al. (2019). Copyright 2019 American Chemical Society.

Stripping the surface ligands of NCs is a direct way to expose more active sites because it can reduce or even eliminate the steric hindrance of ligands (Zhao et al., 2018). However, access to ligand-free NCs is considerably more complicated and constrained by their strong inclination to aggregate (Das et al., 2014; Wang et al., 2015). Exfoliating the surface ligands of NCs encapsulated in MOFs to yield non-ligated NCs is a feasible route, by which the stiff pores of MOFs are capable of preventing the migration of non-ligated NCs, thus maintaining the cluster atomicity. Lately, Fischer and co-workers demonstrated a proof-of-concept by encapsulating CO-ligated, atom-precise Pt_9_ clusters (formula: [NBu_4_]_2_[{Pt_3_(CO)_6_}_3_]) into ZIF-8 and subsequently decarbonylating to generate Pt_12±x_ NCs uniformly distributed in ZIF-8 matrix (Figure 5A) (Kratzl et al., 2019). [NBu_4_]_2_[{Pt_3_(CO)_6_}_3_] would be selectively oxidized into [NBu_4_]_2_[{Pt_3_(CO)_6_}_4_] after being enclosed in ZIF-8 and exposed to air, forming the air-stable inclusion compound [NBu_4_]_2_[{Pt_3_(CO)_6_}_4_]@ZIF-8 of defined atomicity Pt_12_. Whereafter, the [NBu_4_]_2_[{Pt_3_(CO)_6_}_4_]@ZIF-8 composite was heated at 200°C under vacuum to thoroughly remove the carbonyl ligands (Figure 5C). The obtained ligand-free Pt NCs were homogeneously distributed throughout ZIF-8 and showed a diameter of ca. 1 nm, consistent with the cluster size of [NBu_4_]_2_[{Pt_3_(CO)_6_}_4_]@ZIF-8 before decarbonylation (Figure 5B). These results indicated that ZIF-8 matrix can stabilize the decarbonylated Pt NCs against aggregation and therefore maintain the cluster atomicity. Profiting from more exposed metal sites and less diffusion resistance to reactants, the ligand-free Pt NCs in ZIF-8 can fully convert 1-hexene to hexane within 3 h with a TOF of around 2000 h^−1^, much higher than the ligated [NBu_4_]_2_[{Pt_3_(CO)_6_}_4_]@ZIF-8 with only 24% conversion after the same reaction time (Figure 5D).

**Figure 6:**
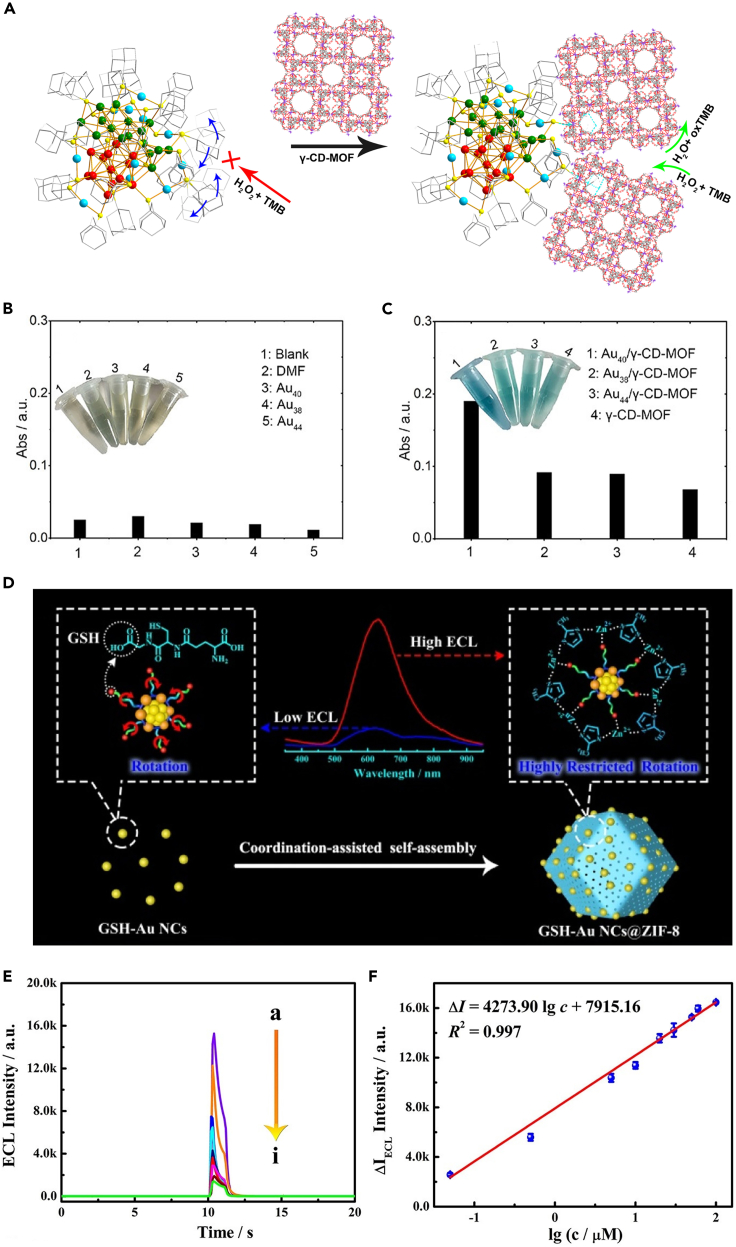
Modulation of functionallity of NCs/MOF composites (A) Schematic representation of the HRP-mimicking reaction catalyzed by Au_40_(S-Adm)_22_ itself (prohibited) and its inclusion compound (permitted). (B and C) Histogram of the absorbance at 652 nm for TMB/H_2_O_2_-related systems (here 3,3′,5,5′-tetramethylbenzidine (TMB) is the substrate) with the addition of different catalysts (in the blank experiment, only TMB and H_2_O_2_ were added). Reproduced with permission from Zhao et al. (2020b). Copyright 2020 American Chemical Society. (D) Schematic representation of the preparation of GSH-Au NCs@ZIF-8 via coordination-assisted self-assembly strategy and the electrochemiluminescence-enhanced mechanism. (E) Electrochemiluminescence intensity of the biosensor with different concentrations of rutin (a) 0.05, (b) 0.5, (c) 5, (d) 10, (e) 20, (f) 30, (g) 50, (h) 60, and (i) 100 μM. (F) Corresponding calibration curve for the rutin determination. Reproduced with permission from Nie et al. (2021). Copyright 2021 American Chemical Society.

Restricting the vibration and rotation of ligands by MOFs is another elegant way to weaken the steric hindrance of ligands, so as to increase the exposure of active sites. Recently, Zhao et al. used a stiff γ-cyclodextrin-MOF (γ-CD-MOF) with adequate cavities to include the ligands of Au_40_(Adm)_22_ NCs by using host-guest chemistry (Figure 6A) (Zhao et al., 2020b). After being anchored on the surface of γ-CD-MOF, each Au_40_(Adm)_22_ is enclosed by approximately 228 γ-CDs and therefore rigidifying the ligands of NCs. As the free-swinging of ligands is impeded, reactants can easily reach and interact with the metal surface of Au_40_ NCs through the interstices left by the rigid ligands. Hence, in a horseradish peroxidase-mimicking reaction, Au_40_/γ-CD-MOF exhibited a higher catalytic activity that the virgin Au_40_ NCs (Figures 6B and 6C). This method is adaptable for other NCs with different ligands such as Au_38_(PET)_24_ and Au_44_(2,4-DMBT)_26_. In another study, Yuan and co-workers used the ligand of NCs as one of the building units of MOFs to impede the ligand swinging of NCs (Nie et al., 2021). Glutathione (GSH) protected Au NCs (GS-Au NCs) were encapsulated into ZIF-8 to construct the GS-Au NCs@ZIF-8 composite, where Zn^2+^ coordinates with both the carboxyl group in the GSH ligand and the nitrogen atom of 2-methylimidazole linkers (Figure 6D). As a consequence, the molecular vibration and rotation of GSH ligands were restrained by the ZIF-8 matrix, as evidenced by the enhanced PL and the increased average lifetime of PL for GS-Au NCs@ZIF-8. Coordinatively rigidifying the GSH ligands with MOF hindered the ligand rotation-induced energy dissipation of Au NCs and diminished the self-quenching effect, generating a significantly improved electrochemiluminescence (ECL) efficiency. Hence, GS-Au NCs@ZIF-8 showed an exceptional sensitivity in the ECL detection of rutin, with a detection limit of 10 nM, surpassing many other biosensors (Figures 6E and 6F).

**Figure 7:**
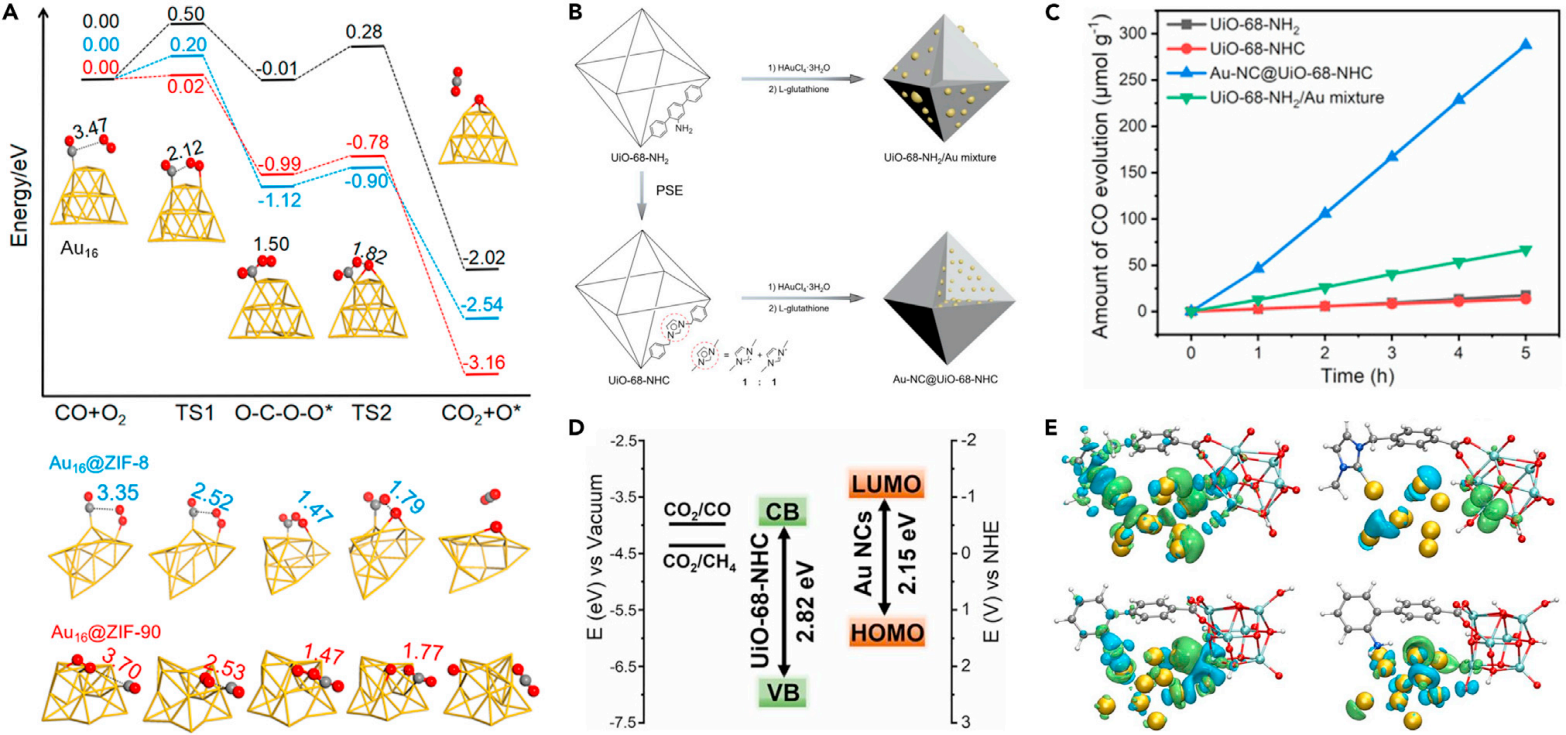
Catalytic application of NCs/MOF composites (A) Energy profiles of CO oxidation using isolated Au_16_ (black line), Au_16_@ZIF-8 (red line), and Au_16_@ZIF-90 (blue line) as catalysts, respectively. Reproduced with permission from Dou et al. (2018). Copyright 2018 American Chemical Society. (B) Schematic representation of the synthesis of UiO-68-NHC, Au-NC@UiO-68-NHC, and UiO-68-NH_2_/Au mixture. (C) Time courses of CO evolution by photocatalytic CO_2_ reduction using UiO-68-NHC, Au-NC@UiO-68-NHC, UiO-68-NH_2_, and Au/UiO-68-NH_2_ as photocatalysts upon AM 1.5G irradiation. (D) Band alignment of Au NCs and UiO-68-NHC. (E) The charge density difference (CDD) under the ground state (left) and the excited state (right) between Au NC (gold) and UiO-68-NHC (top) and UiO-68-NH_2_ (bottom). Electrons and holes are represented by green and blue ellipses, respectively. Reproduced with permission from Jiang et al. (2021). Copyright 2021 Wiley-VCH.

MOFs with well-defined and customizable porous structures are an excellent platform for regulating the microenvironment around the embedded NCs. The interaction between the microenvironment and NCs has a profound influence on the geometric and electronic structures of NCs, which in turn influences the catalytic properties. For example, Dou et al. theoretically investigated the electronic properties of Au NCs encapsulated in ZIF-8 and ZIF-90, as well as their difference in CO oxidation (Dou et al., 2018). The calculation indicated that the different functional groups in ZIF-8 (methyl group) and ZIF-90 (aldehyde group) result in distinct electrical characteristics of the confined Au NCs. Compared with Au NCs confined in ZIF-90, the binding energy of Au NCs inside ZIF-8 is stronger, accompanied with a larger charge transfer from the MOF framework to Au NCs. Furthermore, CO adsorption and oxidation analyses reveal that Au NCs embedded in both ZIF-8 and ZIF-90 have lower reaction barriers than those for isolated Au NCs (Figure 7A), demonstrating the critical function of the MOF support in improving the catalytic activity.

In a recent work, Fei and co-workers utilized an *N*-heterocyclic carbenes (NHCs)-functionalized UiO-68-NH_2_ matrix to immobilize Au NCs for the photocatalytic CO_2_ reduction (Figure 7B) (Jiang et al., 2021). Under illumination, Au-NCs@UiO-68-NHC steadily converted CO_2_ into CO at a rate of 57.6 μmol g^−1^h^−1^, which is over four times higher than the UiO-68-NH_2_/Au mixture without NHC functionalization (13.4 μmol g^−1^h^−1^) (Figure 7C). The dramatic difference in catalytic activity was caused by the different levels of photogenerated electron transfer. For Au-NCs@UiO-68-NHC composite, the photogenerated electrons from Au could be more easily transferred to the Zr_6_O_4_(OH)_4_ clusters of UiO-68-NHC through the Au-NHC covalent bonding because the conduction band of UiO-68-NHC is more positive than the Fermi level of Au NCs (Figure 7D). In contrast, the carrier transfer between Au NCs and UiO-68-NH_2_ is largely limited by their non-covalent interface interactions (Figure 7E).

**Figure 8:**
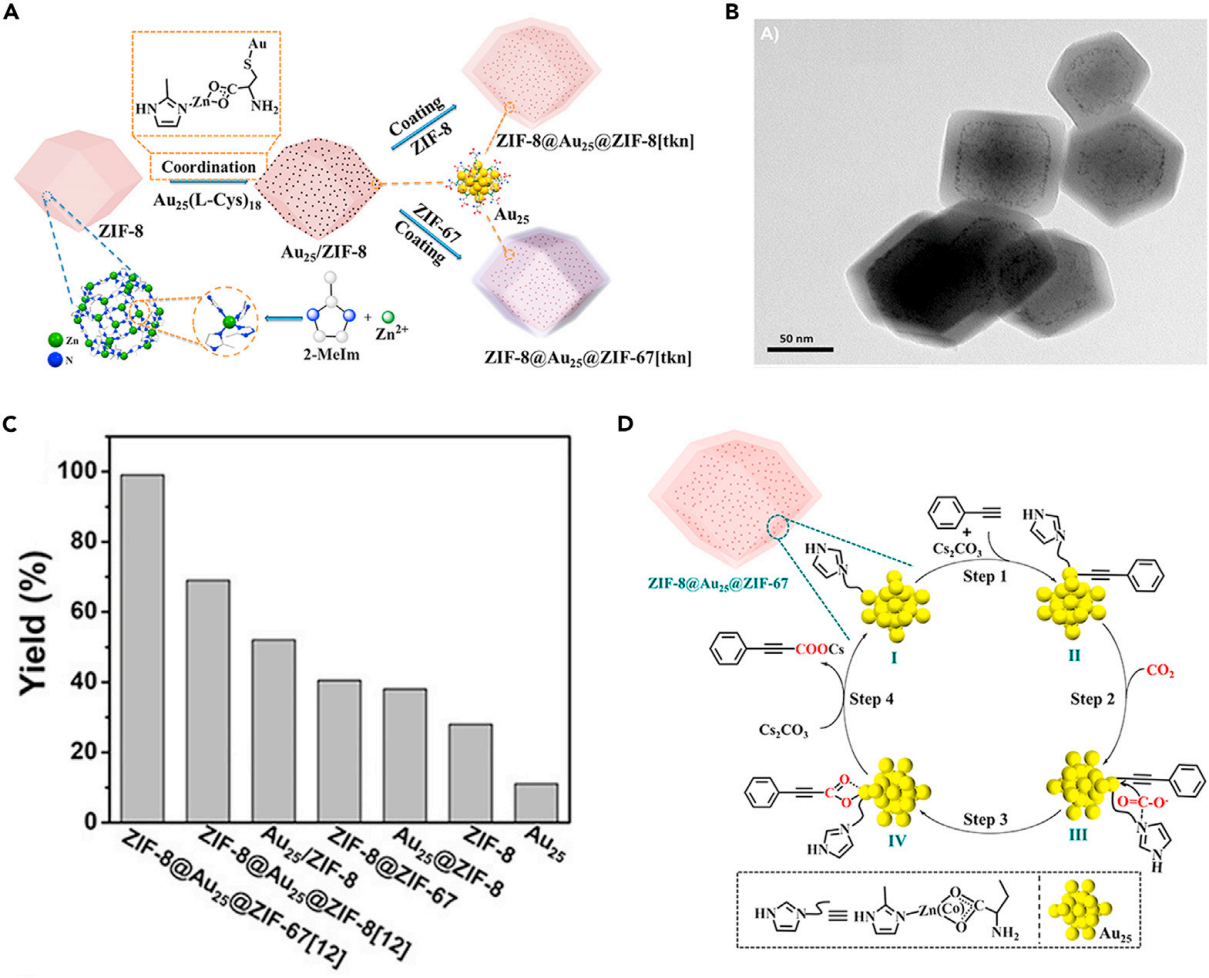
Construction and characterization of AuNCs/ZIF composites (A) Schematic representation of the synthetic route for ZIF-8@Au_25_@ZIF-67 with sandwich structures. (B) TEM images of ZIF-8@Au_25_@ZIF-67 (shell thickness is 12 nm). (C) Activity of various catalysts for the carboxylation of phenylacetylene. (D) Proposed catalytic mechanism of the reaction between terminal alkynes and CO_2_ over ZIF-8@Au_25_@ZIF-67. Reproduced with permission from Yun et al. (2020). Copyright 2020 American Chemical Society.

In addition to altering the structure of NCs, the adjustable microenvironment is also capable of regulating the adsorption and orientation of reactant molecules on catalyst surfaces to synergistically enhance catalytic performance. For example, Zhu and co-workers encapsulated Au_25_(L-Cys)_18_ NCs into the interlayer between ZIF-8 and ZIF-67 to fabricate a robust sandwich-like ZIF-8@Au_25_@ZIF-67 composite for the terminal alkyne carboxylation with CO_2_ (Figures 8A and 8B) (Yun et al., 2020). Beyond accelerating mass transfer and stabilizing Au NCs, the ZIF-67 shell of the sandwich ZIF-8@Au_25_@ZIF-67 does assist capture and activate CO_2_ molecules. In a typical carboxylation process, CO_2_ is captured and activated by 2-methylimidazole of the ZIF-67 shell to form an intermediate state. Then, the activated CO_2_ inserts into the CH≡CH---Au bond for the subsequent catalytic process (Figure 8D). As a result, the sandwich ZIF-8@Au_25_@ZIF-67 displayed the highest activity with a TON value of 4,433 compared with all the other catalysts (Figure 8C). Besides the above functions, the topological structure of porous MOF support can endow the internal NCs with molecule size-selective reactivity.

**Figure 9:**
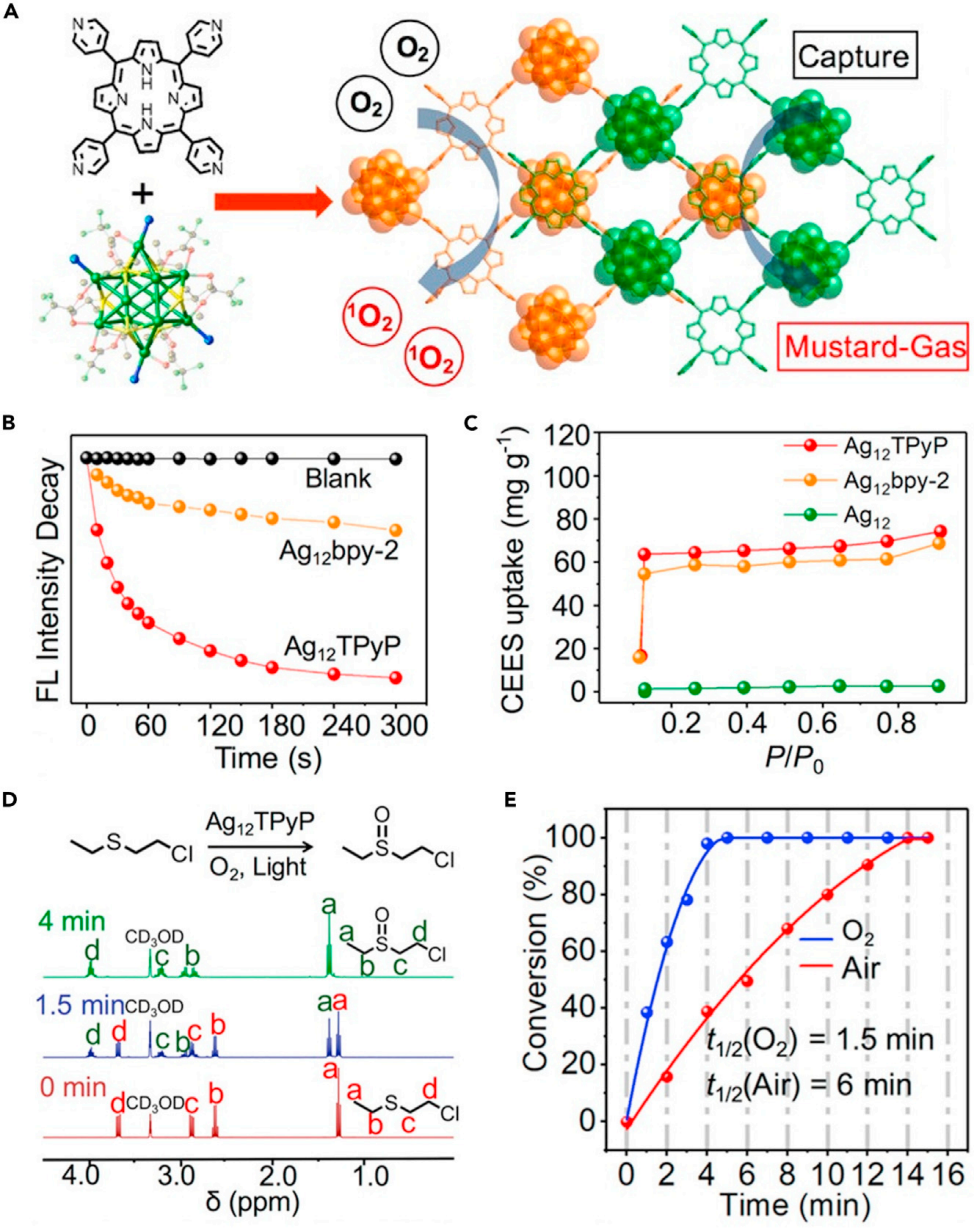
Construction and characterization of AgNCs/MOF composites (A) Schematic representation of the capture and photodetoxification of CEES by Ag_12_TPyP. (B) Degradation of DMA using Ag_12_TPyP and Ag_12_bpy as monitored by the emission decay at 430 nm. DMA acts as the ^1^O_2_ indicator to validate the ^1^O_2_ generation ability of different catalysts. (C) CEES uptake isotherms at 298 K of Ag_12_, Ag_12_TPyP, and Ag_12_bpy. (D) ^1^H NMR analysis of the CEES photooxidation reaction by Ag_12_TPyP. (E) Conversion of CEES in the presence of Ag_12_TPyP under O_2_ (in blue) and air (in red). Reproduced with permission from Cao et al. (2019). Copyright 2019 American Chemical Society.

Unlike incorporating NC guests into MOF hosts, the inter-connection of NCs into the cluster-based MOFs would establish a tight association between the NC building blocks and the bridging linkers, which helps amplify the synergistic effects between the two components for optimizing the catalytic property of NCs. In a typical case, the photosensitizer 5,10,15,20-tetra(4-pyridyl)porphyrin (TPyP) was used as the bridging linker to assemble the Ag_12_ NCs into a new cluster-based MOFs [Ag_12_(S^t^Bu)_6_(CF_3_COO)_3_(TpyP)]_*n*_ (denoted as Ag_12_TpyP) for the effective detoxication of sulfur mustard simulants (2-chloroethyl ethyl sulfide, CEES) (Figure 9A) (Cao et al., 2019). In the network of Ag_12_bpy, the small band gap of Ag_12_ NCs and the TpyP linkers boosted the production efficiency of singlet oxygen (^1^O_2_) under light irradiation (Figure 9B). Meanwhile, owing to (1) the porous frameworks that allow the preconcentration of CEES and (2) the strong affinity between Ag and the chlorine atoms of CEES, Ag_12_TPyP displayed a high capture amount of CEES vapor (74.2 mg g^−1^) (Figure 9C). As a consequence, Ag_12_TPyP demonstrated a superior detoxication efficiency, converting 98% of CEES into the harmless compound CEESO in 4 min with 100% selectivity (Figures 9D and 9E). The synergistic efficiency between the NCs and the TPyP moieties enables Ag_12_TPyP to be an excellent candidate for CEES detoxication compared with the reported MOF-based photocatalysts. Apart from the promotion of catalytic performance, the synergy within the cluster-based MOFs produces unanticipated PL properties, providing a promising platform for the detection of solvents and volatile organic substances. Here we shall not go into depth about the topic of PL enhancement and application since some articles have covered it (Jin et al., 2021b; Kang and Zhu, 2019; Zheng and Xie, 2020).

## Conclusion and perspectives

As summarized above, significant achievements have been made in the combination of NCs and MOFs in recent years. Generally, surface anchoring, pore-confined growth, and *in situ* encapsulation are the three primary synthetic methods for integrating NC guests into MOF hosts. These three techniques are able to unite NCs with MOF in different forms including anchoring NCs on the MOF surface or confining NCs inside MOF. In addition, NCs as building blocks could be assembled into the cluster-based MOFs through the inter-cluster linking procedure, which is an intriguing way to fuse NCs into MOF frameworks.

Owing to the tunable structure and well-defined porosity, MOFs show a great capability in addressing the issues of NCs in catalysis under harsh conditions. First, the rigid frameworks of MOF can effectively prevent the migration and thus aggregation of the embedded NCs. Second, MOFs can increase the accessibility of active sites on NCs surface via reducing the steric hindrance from ligands. Third, the synergy within NC/MOF composites and cluster-based MOFs has a significant impact on the electronic structures of NCs and the adsorption of reactant molecules on NCs surface, both of which are crucial for enhancing the catalytic performance. Last but not least, MOF matrices can endow the embedded NCs with extra distinctive features such as molecular sieving effects.

The development of NCs/MOF composites is quite promising. Below we provide some other perspectives for future efforts.

(1) The encapsulation of NCs into MOFs is still limited to a few specific types like ZIF-67 and ZIF-8. This is because the encapsulation method necessitates that the NCs guests do not interfere with MOF crystal growth and are compatible with the synthesis conditions of MOFs. The solvent-assisted linker exchange of the MOF framework may open up new opportunities. During a typical linker exchange process, the dissociation and association of linkers can temporarily enlarge the pore aperture size to facilitate the diffusion and filling of bigger guests (Li et al., 2018b). This aperture-opening reaction exists in a large number of MOFs and can be controlled by using different solvents. We believe that it has the potential to bring forth some interesting new developments in future work.
(2) The surface ligand of NCs is a double-edged sword. The ligands often suppress the catalytic activity, but their presence protects the NCs and also permits control over the charge distribution between the metal core and the ligands, which can give rise to highly active NCs catalysts for certain reactions. For example, the ligand-capped Au_34_Ag_28_ NCs displayed far better activity in the hydrolytic oxidation of triethylsilane than the related NCs with surface ligands partially or completely removed (Wang et al., 2016). Therefore, the presence of ligands or not may require an optimization for achieving desired catalytic reactivity (Takano et al., 2018). Further effort can be made by the ligand “surgery” of NCs trapped inside MOFs to obtain a site-specific and quantitative removal of ligands, where MOF matrices can stabilize such NCs.
(3) Although some studies have revealed the significant influence of MOF hosts on the properties of NC guests, there are still many interesting questions that need to be addressed. For example, is it possible that the structure of NCs trapped within MOF matrices might experience specific dynamic changes at increased temperatures? How do the ligands progressively detach from the surface of NCs with increasing temperature? In view of the definite structures of both NCs and MOFs, such aspects could be carefully studied by developing real-time, real-space characterization techniques such as high-resolution imaging and spectroscopy (Pollitt et al., 2020). The development and combination of *in situ* techniques would be highly valuable to monitor the catalyst's change and understand the reaction mechanism.
(4) The well-defined and ligand-free NCs are greatly desired for the highest catalytic activity (Jin et al., 2021a; Shivhare et al., 2013; Fang et al., 2016; Kawawaki et al., 2021). Although ligand-free NCs can be obtained by reducing the pre-impregnated precursors of NCs inside MOFs channel, the atomic precision cannot be guaranteed because the preparation of atomically precise NCs involves the kinetically controlled reduction followed by size-focusing or a separation process. Access to the atomically precise NCs without ligands may be accomplished via removing the surface ligands of pre-embedded NCs within MOFs, considering that the rigid pores of MOFs can restrain the migration of “naked” NCs. It is possible that the structure of NCs may undergo specific dynamic changes during the ligand stripping process, but it also provides an opportunity for fine structural control by taking advantage of the customizable pore environments of MOFs.
(5) MOFs are also a type of promising candidates for enhancing the PL efficiency of NCs in addition to optimizing their catalytic capabilities. Once firmly confined within MOF scaffolds, on the one hand, the energy waste (nonradiative decay) caused by unconstrained ligand motion of NCs may be reduced; on the other hand, the self-quenching effect of inter-NCs will be minimized, leading to increased PL efficiency. Furthermore, owing to the direct linkage between NCs nodes and configurable bridging ligands, the cluster-based MOFs showed higher controllability in terms of PL properties. Unfortunately, there are only a few MOFs for encapsulating NCs and only a few types of bridging ligands for inter-cluster connection, making systematic exploration of PL properties of NCs/MOF composites difficult. Thus, NCs/MOF composites for PL applications require a thorough investigation in future work.
(6) Up to now, tens of metal NCs have been created (Jin et al., 2016; Zhou et al., 2019), and more than 20,000 MOFs have been discovered, such as chiral MOFs (Yoon et al., 2012), electrically conductive MOFs (Xie et al., 2020), and luminescent MOFs (Liu et al., 2020). These functionalized MOFs have been employed to optimize the functionalities of various guests for different applications. However, concerning NCs, MOFs are mainly utilized to stabilize the NCs. We believe that the integration of NCs with MOFs of different types will create more functional materials based on each component's intriguing characteristics. More work is warranted to pursue this promising direction.

## References

[bib1] Aguilera-Sigalat J., Bradshaw D. (2016). Synthesis and applications of metal-organic framework-quantum dot (QD@MOF) composites. Coord. Chem. Rev..

[bib2] Alhilaly M.J., Huang R.W., Naphade R., Alamer B., Hedhili M.N., Emwas A.H., Maity P., Yin J., Shkurenko A., Mohammed O.F. (2019). Assembly of atomically precise silver nanoclusters into nanocluster-based frameworks. J. Am. Chem. Soc..

[bib3] Cao M., Pang R., Wang Q.Y., Han Z., Wang Z.Y., Dong X.Y., Li S.F., Zang S.Q., Mak T.C.W. (2019). Porphyrinic silver cluster assembled material for simultaneous capture and photocatalysis of mustard-gas simulant. J. Am. Chem. Soc..

[bib4] Chang Z., Jing X., He C., Liu X., Duan C. (2018). Silver clusters as robust nodes and *π*-activation sites for the construction of heterogeneous catalysts for the cycloaddition of propargylamines. ACS Catal..

[bib5] Chen Y., Zeng C., Kauffman D.R., Jin R. (2015). Tuning the magic size of atomically precise gold nanoclusters via isomeric methylbenzenethiols. Nano Lett..

[bib6] Chen H., Li Z., Qin Z., Kim H.J., Abroshan H., Li G. (2019). Silica-encapsulated gold nanoclusters for efficient acetylene hydrogenation to ethylene. ACS Appl. Nano Mater..

[bib7] Chen Z., Hanna S.L., Redfern L.R., Alezi D., Islamoglu T., Farha O.K. (2019). Reticular chemistry in the rational synthesis of functional zirconium cluster-based MOFs. Coord. Chem. Rev..

[bib8] Das S., Goswami A., Hesari M., Al-Sharab J.F., Mikmeková E., Maran F., Asefa T. (2014). Reductive deprotection of monolayer protected nanoclusters: an efficient route to supported ultrasmall au nanocatalysts for selective oxidation. Small.

[bib9] Deng H., Grunder S., Cordova K.E., Valente C., Furukawa H., Hmadeh M., Gándara F., Whalley A.C., Liu Z., Asahina S. (2012). Large-pore apertures in a series of metal-organic frameworks. Science.

[bib10] Deng G., Teo B.K., Zheng N. (2021). Assembly of chiral cluster-based metal-organic frameworks and the chirality memory effect during their disassembly. J. Am. Chem. Soc..

[bib11] Dong X.-Y., Huang H.-L., Wang J.-Y., Li H.-Y., Zang S.-Q. (2018). A flexible fluorescent Scc-Mof for switchable molecule identification and temperature display. Chem. Mater..

[bib12] Dong X.Y., Si Y., Yang J.S., Zhang C., Han Z., Luo P., Wang Z.Y., Zang S.Q., Mak T.C.W. (2020). Ligand engineering to achieve enhanced ratiometric oxygen sensing in a silver cluster-based metal-organic framework. Nat. Commun..

[bib13] Dou L., Wu S., Chen D.-L., He S., Wang F.-F., Zhu W. (2018). Structures and electronic properties of au clusters encapsulated ZIF-8 and ZIF-90. J. Phys. Chem. C.

[bib14] Du X.S., Yan B.J., Wang J.Y., Xi X.J., Wang Z.Y., Zang S.Q. (2018). Layer-sliding-driven crystal size and photoluminescence change in a novel Scc-MOF. Chem. Commun..

[bib15] Du Y., Sheng H., Astruc D., Zhu M. (2020). Atomically precise noble metal nanoclusters as efficient catalysts: a bridge between structure and properties. Chem. Rev..

[bib16] Falcaro P., Ricco R., Yazdi A., Imaz I., Furukawa S., Maspoch D., Ameloot R., Evans J.D., Doonan C.J. (2016). Application of metal and metal oxide nanoparticles@MOFs. Coord. Chem. Rev..

[bib17] Fang J., Zhang B., Yao Q., Yang Y., Xie J., Yan N. (2016). Recent advances in the synthesis and catalytic applications of ligand-protected, atomically precise metal nanoclusters. Coord. Chem. Rev..

[bib18] Fenlon E.E. (2018). What tangled webs we weave. Nat. Chem..

[bib19] Gao Q., Xu S., Guo C., Chen Y., Wang L. (2018). Embedding nanocluster in MOF via crystalline ion-triggered growth strategy for improved emission and selective sensing. ACS Appl. Mater. Inter..

[bib20] Gunawardene P.N., Corrigan J.F., Workentin M.S. (2019). Golden opportunity: a clickable azide-functionalized [Au_25_(SR)_18_]^−^ nanocluster platform for interfacial surface modifications. J. Am. Chem. Soc..

[bib21] Higaki T., Li Q., Zhou M., Zhao S., Li Y., Li S., Jin R. (2018). Toward the tailoring chemistry of metal nanoclusters for enhancing functionalities. Acc. Chem. Res..

[bib22] Huang Y.B., Liang J., Wang X.S., Cao R. (2017). Multifunctional metal-organic framework catalysts: synergistic catalysis and tandem reactions. Chem. Soc. Rev..

[bib23] Huang R.W., Wei Y.S., Dong X.Y., Wu X.H., Du C.X., Zang S.Q., Mak T.C.W. (2017). Hypersensitive dual-function luminescence switching of a silver-chalcogenolate cluster-based metal-organic framework. Nat. Chem..

[bib24] Huang R.W., Dong X.Y., Yan B.J., Du X.S., Wei D.H., Zang S.Q., Mak T.C.W. (2018). Tandem silver cluster isomerism and mixed linkers to modulate the photoluminescence of cluster-assembled materials. Angew. Chem. Int. Ed..

[bib25] Jiang Y., Yu Y., Zhang X., Weinert M., Song X., Ai J., Han L., Fei H. (2021). N-heterocyclic carbene-stabilized ultrasmall gold nanoclusters in a metal-organic framework for photocatalytic CO_2_ reduction. Angew. Chem. Int. Ed..

[bib26] Jin R., Zeng C., Zhou M., Chen Y. (2016). Atomically precise colloidal metal nanoclusters and nanoparticles: fundamentals and opportunities. Chem. Rev..

[bib27] Jin R., Li G., Sharma S., Li Y., Du X. (2021). Toward active-site tailoring in heterogeneous catalysis by atomically precise metal nanoclusters with crystallographic structures. Chem. Rev..

[bib28] Jin Y., Zhang C., Dong X.Y., Zang S.Q., Mak T.C.W. (2021). Shell engineering to achieve modification and assembly of atomically-precise silver clusters. Chem. Soc. Rev..

[bib29] Kang X., Zhu M. (2019). Intra-cluster growth meets inter-cluster assembly: the molecular and supramolecular chemistry of atomically precise nanoclusters. Coord. Chem. Rev..

[bib30] Kawawaki T., Kataoka Y., Hirata M., Iwamatsu Y., Hossain S., Negishi Y. (2021). Toward the creation of high-performance heterogeneous catalysts by controlled ligand desorption from atomically precise metal nanoclusters. Nanoscale Horizons.

[bib31] Kirchon A., Feng L., Drake H.F., Joseph E.A., Zhou H.C. (2018). From fundamentals to applications: a toolbox for robust and multifunctional MOF materials. Chem. Soc. Rev..

[bib32] Kratzl K., Kratky T., Gunther S., Tomanec O., Zboril R., Michalicka J., Macak J.M., Cokoja M., Fischer R.A. (2019). Generation and stabilization of small platinum clusters Pt_12±x_ inside a metal-organic framework. J. Am. Chem. Soc..

[bib33] Li G., Jin R. (2013). Atomically precise gold nanoclusters as new model catalysts. Acc. Chem. Res..

[bib34] Li Y., Jin R. (2020). Seeing ligands on nanoclusters and in their assemblies by x-ray crystallography: atomically precise nanochemistry and beyond. J. Am. Chem. Soc..

[bib35] Li G., Abroshan H., Liu C., Zhuo S., Li Z., Xie Y., Kim H.J., Rosi N.L., Jin R. (2016). Tailoring the electronic and catalytic properties of Au_25_ nanoclusters via ligand engineering. ACS Nano.

[bib36] Li Y., Chen Y., House S.D., Zhao S., Wahab Z., Yang J.C., Jin R. (2018). Interface engineering of gold nanoclusters for CO oxidation catalysis. ACS Appl. Mater. Inter..

[bib37] Li Z., Rayder T.M., Luo L., Byers J.A., Tsung C.-K. (2018). Aperture-opening encapsulation of a transition metal catalyst in a metal-organic framework for CO_2_ hydrogenation. J. Am. Chem. Soc..

[bib39] Liu C., Zeng C., Luo T.Y., Merg A.D., Jin R., Rosi N.L. (2016). Establishing porosity gradients within metal-organic frameworks using partial postsynthetic ligand exchange. J. Am. Chem. Soc..

[bib40] Liu L., Song Y., Chong H., Yang S., Xiang J., Jin S., Kang X., Zhang J., Yu H., Zhu M. (2016). Size-confined growth of atom-precise nanoclusters in metal-organic frameworks and their catalytic applications. Nanoscale.

[bib38] Li Y., Zhou M., Jin R. (2021). Programmable metal nanoclusters with atomic precision. Adv. Mater..

[bib41] Liu X.-Y., Lustig W.P., Li J. (2020). Functionalizing luminescent metal-organic frameworks for enhanced photoluminescence. ACS Energy Lett.

[bib42] Longo A., Boed E.J.J., Mammen N., Linden M., Honkala K., Häkkinen H., Jongh P.E., Donoeva B. (2020). Towards atomically precise supported catalysts from monolayer-protected clusters: the critical role of the support. Chem. Eur. J..

[bib43] Luo Y., Fan S., Yu W., Wu Z., Cullen D.A., Liang C., Shi J., Su C. (2018). Fabrication of Au_25_(SG)_18_-ZIF-8 nanocomposites: a facile strategy to position Au_25_(SG)_18_ nanoclusters inside and outside ZIF-8. Adv. Mater*.*.

[bib44] Ma X.H., Wang J.Y., Guo J.J., Wang Z.Y., Zang S.Q. (2019). Reversible wide-range tuneable luminescence of a dual-stimuli- responsive silver cluster-assembled. Mater. Chin. J. Chem..

[bib45] Nie Y., Tao X., Zhang H., Chai Y.Q., Yuan R. (2021). Self-assembly of gold nanoclusters into a metal-organic framework with efficient electrochemiluminescence and their application for sensitive detection of rutin. Anal. Chem..

[bib46] Pan Y., Qian y., Zheng X., Chu S.-Q., Yang Y., Ding C., Wang X., Yu S.-H., Jiang H.-L. (2021). Precise fabrication of single-atom alloy co-catalyst with optimal charge state for enhanced photocatalysis. Natl. Sci. Rev..

[bib47] Pollitt S., Truttmann V., Haunold T., Garcia C., Olszewski W., Llorca J., Barrabés N., Rupprechter G. (2020). The dynamic structure of Au_38_(SR)_24_ nanoclusters supported on CeO_2_ upon pretreatment and CO oxidation. ACS Catal..

[bib48] Rogge S.M.J., Bavykina A., Hajek J., Garcia H., Olivos-Suarez A.I., Sepulveda-Escribano A., Vimont A., Clet G., Bazin P., Kapteijn F. (2017). Metal-organic and covalent organic frameworks as single-site catalysts. Chem. Soc. Rev..

[bib49] Shivhare A., Chevrier D.M., Purves R.W., Scott R.W.J. (2013). Following the thermal activation of Au_25_(SR)_18_ clusters for catalysis by x-ray absorption spectroscopy. J. Phys. Chem. C.

[bib50] Sun L., Yun Y., Sheng H., Du Y., Ding Y., Wu P., Li P., Zhu M. (2018). Rational encapsulation of atomically precise nanoclusters into metal-organic frameworks by electrostatic attraction for CO_2_ conversion. J. Mater. Chem. A..

[bib51] Takano S., Hasegawa S., Suyama M., Tsukuda T. (2018). Hydride doping of chemically modified gold-based superatoms. Acc. Chem. Res..

[bib52] Wang Y., Su H., Xu C., Li G., Gell L., Lin S., Tang Z., Häkkinen H., Zheng N. (2015). An intermetallic Au_24_Ag_20_ superatom nanocluster stabilized by labile ligands. J. Am. Chem. Soc*.*.

[bib53] Wang Y., Wan X.K., Ren L., Su H., Li G., Malola S., Lin S., Tang Z., Hakkinen H., Teo B.K. (2016). Atomically precise alkynyl-protected metal nanoclusters as a model catalyst: observation of promoting effect of surface ligands on catalysis by metal nanoparticles. J. Am. Chem. Soc..

[bib54] Wang Z.Y., Wang M.Q., Li Y.L., Luo P., Jia T.T., Huang R.W., Zang S.Q., Mak T.C.W. (2018). Atomically precise site-specific tailoring and directional assembly of superatomic silver nanoclusters. J. Am. Chem. Soc..

[bib55] Xie L.S., Skorupskii G., Dinca M. (2020). Electrically conductive metal-organic frameworks. Chem. Rev..

[bib56] Yan Z., Taylor M.G., Mascareno A., Mpourmpakis G. (2018). Size-, shape-, and composition-dependent model for metal nanoparticle stability prediction. Nano Lett..

[bib57] Yang Q., Xu Q., Jiang H.L. (2017). Metal-organic frameworks meet metal nanoparticles: synergistic effect for enhanced catalysis. Chem. Soc. Rev..

[bib58] Yoon M., Srirambalaji R., Kim K. (2012). Homochiral metal-organic frameworks for asymmetric heterogeneous catalysis. Chem. Rev..

[bib59] Yun Y., Sheng H., Bao K., Xu L., Zhang Y., Astruc D., Zhu M. (2020). Design and remarkable efficiency of the robust sandwich cluster composite nanocatalysts ZIF-8@Au_25_@ZIF-67. J. Am. Chem. Soc..

[bib60] Zhang W., Lu G., Cui C., Liu Y., Li S., Yan W., Xing C., Chi Y.R., Yang Y., Huo F.A. (2014). Family of metal-organic frameworks exhibiting size-selective catalysis with encapsulated noble-metal nanoparticles. Adv. Mater..

[bib61] Zhao J., Li Q., Zhuang S., Song Y., Morris D.J., Zhou M., Wu W., Zhang P., Jin R. (2018). Reversible control of chemoselectivity in Au_38_(SR)_24_ nanocluster-catalyzed transfer hydrogenation of nitrobenzaldehyde derivatives. J. Phys. Chem. Lett..

[bib62] Zhao M., Huang S., Fu Q., Li W., Guo R., Yao Q., Wang F., Cui P., Tung C.H., Sun D. (2020). Ambient chemical fixation of CO_2_ using a robust Ag_27_ cluster-based two-dimensional metal-organic framework. Angew. Chem. Int. Ed..

[bib63] Zhao Y., Zhuang S., Liao L., Wang C., Xia N., Gan Z., Gu W., Li J., Deng H., Wu Z. (2020). A dual purpose strategy to endow gold nanoclusters with both catalysis activity and water solubility. J. Am. Chem. Soc..

[bib64] Zheng K., Xie J. (2020). Engineering ultrasmall metal nanoclusters as promising theranostic agents. Trends Chem..

[bib65] Zhou H.C., Long J.R., Yaghi O.M. (2012). Introduction to metal-organic frameworks. Chem. Rev..

[bib66] Zhou M., Higaki T., Hu G., Sfeir M.Y., Chen Y., Jiang D., Jin R. (2019). Three-orders-of-magnitude variation of carrier lifetimes with crystal phase of gold nanoclusters. Science.

